# Mechanochemical Pretreated M_n+1_AX_n_ (MAX) Phase to Synthesize 2D-Ti_3_C_2_T_x_ MXene Sheets for High-Performance Supercapacitors

**DOI:** 10.3390/nano13111741

**Published:** 2023-05-26

**Authors:** Inho Cho, Aravindha Raja Selvaraj, Jinsoo Bak, Heeje Kim, Kandasamy Prabakar

**Affiliations:** Department of Electrical Engineering, Pusan National University, 2 Busandaehak-ro 63beon-gil, Geumjeong-gu, Busan 46241, Republic of Korea; whdlsgh92@icloud.com (I.C.); rajanano12@gmail.com (A.R.S.); xxximjsn@gmail.com (J.B.); heejekim734@gmail.com (H.K.)

**Keywords:** ball milling, 2D-Ti_3_C_2_Tx MXene, intercalation, supercapacitor

## Abstract

Two-dimensional (2D) MXenes sheet-like micro-structures have attracted attention as an effective electrochemical energy storage material due to their efficient electrolyte/cation interfacial charge transports inside the 2D sheets which results in ultrahigh rate capability and high volumetric capacitance. In this article, Ti_3_C_2_T_x_ MXene is prepared by a combination of ball milling and chemical etching from Ti_3_AlC_2_ powder. The effects of ball milling and etching duration on the physiochemical properties are also explored, as well as the electrochemical performance of as-prepared Ti_3_C_2_ MXene. The electrochemical performances of 6 h mechanochemically treated and 12 h chemically etched MXene (BM-12H) exhibit an electric double layer capacitance behavior with an enhanced specific capacitance of 146.3 F g^−1^ compared to 24 and 48 h treated samples. Moreover, 5000-cycle stability tested sample’s (BM-12H) charge/discharge show increased specific capacitance due to the termination of the -OH group, intercalation of K^+^ ion and transformation to TiO_2_/Ti_3_C_2_ hybrid structure in a 3 M KOH electrolyte. Interestingly, a symmetric supercapacitor (SSC) device fabricated in a 1 M LiPF_6_ electrolyte in order to extend the voltage window up to 3 V shows a pseudocapacitance behavior due to Li on interaction/de-intercalation. In addition, the SSC shows an excellent energy and power density of 138.33 W h kg^−1^ and 1500 W kg^−1^, respectively. The ball milling pre-treated MXene exhibited an excellent performance and stability due to the increased interlayer distance between the MXene sheets and intercalation and deintercalation of Li^+^ ions.

## 1. Introduction

Since the second industrial revolution and until recently, mankind has mainly depended on fossil fuels to produce energy. Energy produced from fossil fuels has caused many side effects such as global warming due to its by-product CO_2_; moreover, the depletion of source materials might cause a great energy crisis in the future [1,2]. Hence, much research interest is focused on eco-friendly, sustainable, and renewable energy production mechanisms [3]. At the same time, energy storage devices also play a crucial role in storing the produced energy, and research interest in these devices also keeps increasing. It is an undeniable fact that the efficient transformation and saving of electrochemical energy is the most important part of the clean energy portfolio. In this respect, fuel cells, batteries, and supercapacitors have been considered as promising electrochemical energy storage devices [4,5,6,7] in the academia and various industries [8].

Supercapacitor is a highly promising future electrochemical energy storage (ESS) device. Even though lithium-ion batteries have the largest ESS market share in the world, their life cycle and the value of power density are limited due to the shuttling of lithium cations (Li^+^) between the electrodes and the phase transformation of the cathode materials [9,10]. Supercapacitors have a long life cycle and high power density; however, they also have low energy density. Hence, it is imperative to narrow the gap between batteries and supercapacitors so as to obtain high energy and power densities for the future ESS devices [11,12,13].

Supercapacitor consists of an electrode, an electrolyte, and a separator. Among them, the electrode material has the greatest influence on the properties of the supercapacitor. Based on these electrode materials, supercapacitors are largely divided into electric double layer capacitors (EDLC) mainly made from carbon materials, pseudo-capacitors made from transition metal oxides, and hybrid supercapacitors using both materials at the individual electrodes [14,15,16]. Each material has distinct advantages and disadvantages. For example, carbon-based materials used in EDLC have excellent power density and cycle life but have limited energy density (less than 10 W h kg^−1^) [17,18]. Transition metal oxides used in pseudo-capacitors have higher energy density; however, they also have low conductivity, and an unstable chemical bonding limits the cycling stability [19,20,21]. As a result, a rational electrode assembly may effectively minimize the effects of electrolyte ion diffusion resistance and improve ionic transport inside the electrode surface. Over the recent decade, a variety of novel nanomaterials belonging to the family of 2D transition metal carbide and/or nitride such as carbonitrides known as MXene have been developed and investigated for SC applications [22,23]. Similar to MXene, graphene with two-dimensional structures has already been reported with unique and excellent properties for various applications [24,25]. MXenes with graphene-like 2D layered structures have boosted the performance of supercapacitors due to its surface redox reactions/intercalation pseudocapacitance charge storage mechanism. However, they also show electric double layer (EDLC) behavior [26,27]. Other distinguishing features include high intrinsic metallic conductivity (2 × 10^5^ S m^−1^) and the presence of abundant surface terminated functional groups such as -F, -OH, -N which are beneficial for surface redox reactions, and their hydrophilicity has proven to be a potential electrode for supercapacitors [28,29].

The M_n+1_AX_n_ phases are generally made of layers of transition metal carbides or nitrides that are interleaved with layers of the A group (e.g., Al, Ga, Si, or Ge) elements; M is an early transition metal, and X is carbon and/or nitrogen [22,26]. The MXenes (M_n+1_X_n_T_x_) are generally produced by selectively etching layers from the parent MAX phases where T_x_ are the surface functional groups (typically =O, −OH, −Cl, and −F) [30,31,32]. The mechanical shearing of MAX phases has been found to be ineffective in separating the M_n+1_X_n_ layers and producing MXenes because the M-A bond is metallic [33,34]. However, the M–A bonds are more chemically dynamic than the stronger M–X bonds, which makes selective etching of the A layers possible [22,35]. The energy storage performance of MXenes can be varied by tuning the interlayer distances of M_n+1_X_n_ layers by effective etching methods [36,37].

One of the most widely studied MXenes materials (Ti_3_C_2_T_x_) has been derived from selective etching of Ti_3_AlC_2_ precursors [38]. The Ti_3_C_2_T_x_-based MXene materials have been widely studied in applications such as solar cells [39,40], electrocatalysis [41,42], energy storage [43,44,45], water conversion [46,47], and sensors [48,49]. The energy storage capability of Ti_3_C_2_T_x_ is mostly determined by aluminum exfoliation, and hence, most studies have been focused on the time and synthesis mechanism of the Al exfoliation process [50,51,52]. Though an enormous amount of work has been conducted on ultrasonication and chemical HF-etching effect, limited work has reported on mechanical milling effect [53]. Ball milling technique is a simple, rapid, easy to scale up and hence is a practical method to realize the large-scale extraction of 2D materials [54,55]. In addition, under the influence of mechanical ball milling, the 2D layered material is exfoliated into microscale/nanoscale sheets [56,57]. For example, graphene, boron nitride, and molybdenum disulfide have all been successfully exfoliated by the ball milling method [34,58]. Mechanical deformation of the MAX phases can stimulate the partial exfoliation of Al layers and could initiate the formation of varied M_n+1_X_n_ layer thicknesses ranging from tens to hundreds of nanometers [59,60]. Similarly, ball milling effect on the MAX phase can induce the reduction in particle size, thereby creating edge defects and porosity which may facilitate easy charge–carrier transport and hence enhance electrochemical performance [60].

Even though many researchers have achieved high specific capacitance on MXenes electrodes, unfortunately, many MXene 2D Ti_3_C_2_T_x_ electrodes show inferior energy density in symmetric supercapacitors due to the lesser operating working voltage < 1 V in aqueous electrolytes which limits the practical application. Hence, we have thoroughly investigated the two-electrode system in an organic electrolyte and achieved high energy densities due to a high operating voltage of 3.0 V.

In this study, we have described a rapid strategy to produce 2D layered MXene sheets from the MAX phase. The proposed approach employs a novel mechanochemical-assisted stirring process to break the aluminium (Al) bond from the MAX phase, which significantly shortens the synthesis period from 48 to 12 h. In addition, we explored the effects of ball milling and etching duration on the crystalline structure, morphology, chemical stability and the electrochemical activity of as-prepared Ti_3_C_2_T_x_ MXene in neutral aqueous electrolyte. In particular, the as-fabricated few-layer Ti_3_C_2_T_x_ possesses a large interlayer spacing, which allows the excellent penetration of electrolyte and short diffusion paths for charge carriers. Thus, the SC shows high specific capacitances, impressive rate capabilities, and long cycling stabilities. To improve the energy density, a symmetric supercapacitor (SSC) coin cell device is fabricated and tested in organic electrolyte.

## 2. Experimental Section

### 2.1. Materials

All chemical reagents were of analytical grade and used as is without further purification. Ti_3_AlC_2_ (MAX, 200 mesh) powder was received from Luoyang Advanced Material Co., Ltd. (Luoyang, China). Ethanol, hydrofluoric acid (HF, concentration 49%), hydrochloric acid (HCl, concentration 35.0–37.0%), potassium hydroxide (KOH), and N-methyl-2-pyrrolidinone (NMP) were purchased from SAMCHUN chemicals, Seoul, Republic of Korea. The poly (vinylidene fluoride) and carbon black were purchased from Sigma Aldrich (St. Louis, MO, USA). Carbon cloth (CC) was obtained from the MTI (Richmond, CA, USA) Corporation.

### 2.2. Experiment

#### 2.2.1. Ball Milling Procedure

The as-received MAX (Ti_3_AlC_2_) powder was grinded by (Retsch, Haan, Germany) a planetary ball mill with a rotation speed of 300 rpm for 6 h. In a typical experiment, 5 g of Ti_3_AlC_2_ and 10 mL of ethanol were poured into a ball milling jar containing 500 g zirconia balls (12 mm in diameter). The milled MAX powder was dried at 80 °C overnight and termed as BMAX-6H.

#### 2.2.2. Preparation of Ti_3_C_2_ MXene

Ti_3_C_2_ MXene was prepared by way of the HF etching process. In particular, 4 g of ball milled Ti_3_AlC_2_ powder was slowly poured into 40 mL of a 49% HF aqueous solution. The suspension was then stirred at 60 °C for 12 h under fume hood. This acidic mixture was diluted in de-ionized water (DI H_2_O) and ethanol, followed by repetitive centrifugation (4000 rpm, 6 min each cycle) until the pH reached ~6, and then dried in an oven at 80 °C for 12 h. For comparison, the received Ti_3_AlC_2_ powders were also etched at different times (24 and 48 h) using the same procedure. The MXene samples were named BM-12H (ball milled/12 h Etching), M-24H (only etched for 24 h), and M-48H (only etched for 48 h) (see Table S1).

### 2.3. Material Characterization

The XRD data in this study were acquired using an X-ray diffractometer (D/max-2400, Rigaku, Tokyo, Japan) via Cu K radiation. Field emission scanning electron microscopy (FESEM, Zeiss Gemmini SEM 500, Oberkochen, Germany) was used to assess the surface morphology. High-resolution transmission electron microscopy (HRTEM, JEM-2100F, 200 kV) was used to analyze the morphologies of MXene. The phase composition of the as-prepared samples was investigated by XPS using a Thermo Fisher Scientific (Swindon, UK) ESCALAB 250 system with monochromatic Al Kα radiation at 1486.6 eV. XPS software (Casa Software Ltd., Devon, England) was used for peak fitting and quantitative analysis. The Brunauer–Emmett–Teller (BET) method was used to analyze the surface area and pore size distribution of the samples with ASAP 2010 (Accelerated Surface Area and Porosimetry System, Norcross, GA, USA) using N2 as probe gas. The SSA was determined by applying the multipoint BET method to obtain the N2 adsorption data for the relative pressures (P/P0) between 0.005 and 0.15. The specific pore volume (SPV), micropore volume, and pore size distribution (PSD) were estimated using the BJH method.

### 2.4. Electrochemical Studies

The electrodes were prepared by mixing the MXene active materials, PVDF and acetylene black at a mass ratio of 85:10:5 in the 1-methyl-2-pyrrolidone (NMP) solvent and then ground using mortar and pestle to form a homogeneous slurry. The electrodes were coated on pre-treated carbon cloth (CC) using standard slurry process, and then dried in an oven at 120 °C for 6 h. The electrode material’s mass loading on a single electrode was approximately 4 mg cm^−2^. The electrochemical performance of the MXene electrodes was assessed by cyclic voltammetry (CV), galvanostatic charge–discharge (GCD), and electrochemical impedance spectroscopy (EIS) using a Biologic 150 electrochemical workstation in a 3 M KOH aqueous electrolyte solution. The electrochemical experiments were conducted in a three-electrode cell assembly to assess the performance of individual electrodes (working electrode) with an Hg/HgO reference electrode and a platinum wire as a counter electrode at ambient temperature.

#### Fabrication of Coin-Type Symmetric Supercapacitors in Organic Electrolytes

Two symmetrical MXene electrodes were inserted in a typical CR2032 coin cell, separated by a porous polypropylene separator (Celgard 3401, Charlotte, NC, USA), and the cell was sealed with 1 M LiPF_6_ in EC/DMC (1:1) electrolyte in an argon-filled glove box. The detailed electrochemical calculations are provided in the supplementary information (SI).

## 3. Results and Discussion

### 3.1. X-ray Diffraction(XRD) Analysis

**Figure 1:**
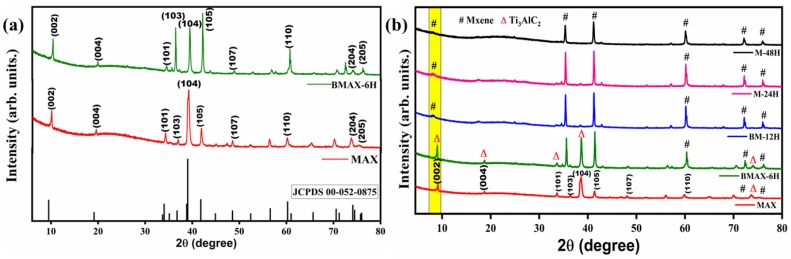
XRD patterns of (**a**) MAX phase and milled MAX phase; (**b**) MAX Phases and MXenes etched at different times.

The XRD (Figure 1) was employed to analyze the phase changes occurring in Ti_3_AlC_2_ during the transformation into Ti_3_C_2_. The diffraction peak pattern of the MAX powder (Ti_3_AlC_2_) along with a 6 h ball milled MAX phase (BMAX-6H) is provided in Figure 1a. Both the MAX powder and BMAX-6H matched well with the Ti_3_AlC_2_ standard diffraction pattern (JCPDS: 00-052-0875), displaying characteristic peaks at 9.54° (002), 19.24° (004), 34.10° (101), 36.7° (103), 39.1° (104), 41.8° (105), 48.53° (107), 60.1(110), 74.5° (204) and 75.8° (205) crystal planes [34,52]. Compared with the pristine Ti_3_AlC_2_, the peak (002) corresponding to Ti_3_C_2_T_x_ was slightly shifted to a lower 2θ = 9.15° value after the ball milling in sample BMAX-6H (Figure 1a) leading to a larger interatomic distance [60]. After the mechanochemical pretreatment, the intensities of Ti_3_C_2_T_x_ diffraction peaks such as (103), (105) and (110) crystal planes were increased. These results clearly imply that the ball milling pretreatment not only helped to enlarge the interlayer distance, but also initiated the exfoliation of Al layers caused by the mechanical stress provided by ball milling. The XRD patterns of BM-12H, M-24H, and M-48H are shown in Figure 1b along with the Ti_3_AlC_2_ phase. After HF chemical etching, MXenes (Ti_3_C_2_T_x_) exhibit sharp peaks of the Ti_3_C_2_ crystal planes with a concomitant decrease in intensity of the Ti_3_AlC_2_ phase (nearly disappeared), suggesting the successful etching of the Al layers. Moreover, all MXenes show a weak and broad peak around 8.43° to 8.61° compared to the initial peak intensity of the parent Ti_3_AlC_2_ phase (002) peak at 9.54°. This corresponds to a 7% increase in the d-spacing along the c-axis, which in turn increases the interlayer distance between the MXene sheets [33,61]. The disappearance of Ti_3_AlC_2_ (002) and (004) peaks clearly indicates the excellent etching effect. It is very interesting to note that irrespective of the etching reaction time, ball milling pretreatment promotes the conversion of Ti_3_AlC_2_ into MXene.

### 3.2. Morphological and Elemental Analysis

Figure S1 displays the FESEM images of the as-received Ti_3_AlC_2_ MAX powders and treated with ball milling. The parent Ti_3_AlC_2_ MAX (Figure S1a,b) demonstrates a distinctive morphology of a stacked layered structure that is closely packed with a smooth surface. Figure S1c shows the layered rigid structure, but the magnified FESEM images (Figure S1c,d) clearly show the cracked, wrinkled, and unsmoothed structure on the edges and the surface of the MAX phase. The mechanical deformation of the MAX phase could lead to partial exfoliation and formation of a layered MXene structure [62]. Therefore, the loosely bound stacked layers formed during the ball milling are easily etched by HF and effectively remove the Al [33].

**Figure 2:**
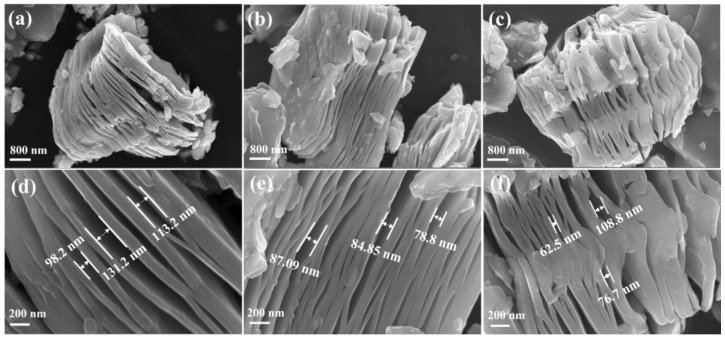
FESEM images of MXene at different HF etching times. (**a**,**d**) BM-12H (**b**,**e**) M-24H and (**c**,**f**) M-48H.

The surface morphologies of the BM-12H, M-24H, and M-48H samples investigated by FESEM are shown in Figure 2a–c. All the etched Ti_3_C_2_T_x_-MXene structures have stacked multilayers due to the removal of Al layers from the MAX phase [52]. In the magnified FESEM images, the 2D layer is uniformly formed in all MXenes with an interlayer distance of 131.2 nm in ball-milled MXene (Figure 2d) compared to the other two MXenes, M-24h (87.09 nm) and M-48h (76.09 nm), which could facilitate the electrolyte penetration during the charging and discharging process with a large surface area [50,52].

**Figure 3:**
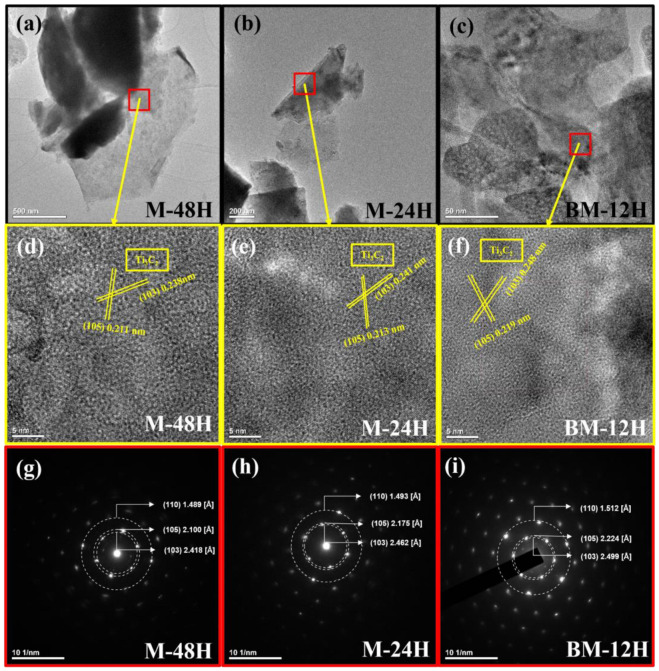
HRTEM images of M-48H (**a**,**d**), M-24H (**b**,**e**), BM-12H (**c**,**f**) in low and high magnification. The red boxes are the designated area for obtaining the SAED pattern of M-48H (**g**), M-24H (**h**), BM-12H (**i**).

Figure 3 shows the HRTEM images and the SAED patterns of the samples. Figure 3a–c shows the low-magnification HRTEM image which clearly evidences the multilayer structure, a representative characteristic of Ti_3_C_2_T_x_ MXene. Figure 3d–f shows the HRTEM images with high magnification where we can observe specific and typical Ti_3_C_2_ lattice patterns representing (103) and (105) crystal planes, which agrees with the XRD [63,64]. Interestingly, the width of the lattice pattern in BM-12H was found to be higher than the others and agree with the SAED pattern shown in Figure 3g–i. The width of the lattice pattern and the D-spacing [Å] values are shown at Table 1. The D-spacing [Å] of BM-12H is higher than that of the other two samples, suggesting a larger interlayer distance.

### 3.3. XPS Analysis

**Figure 4:**
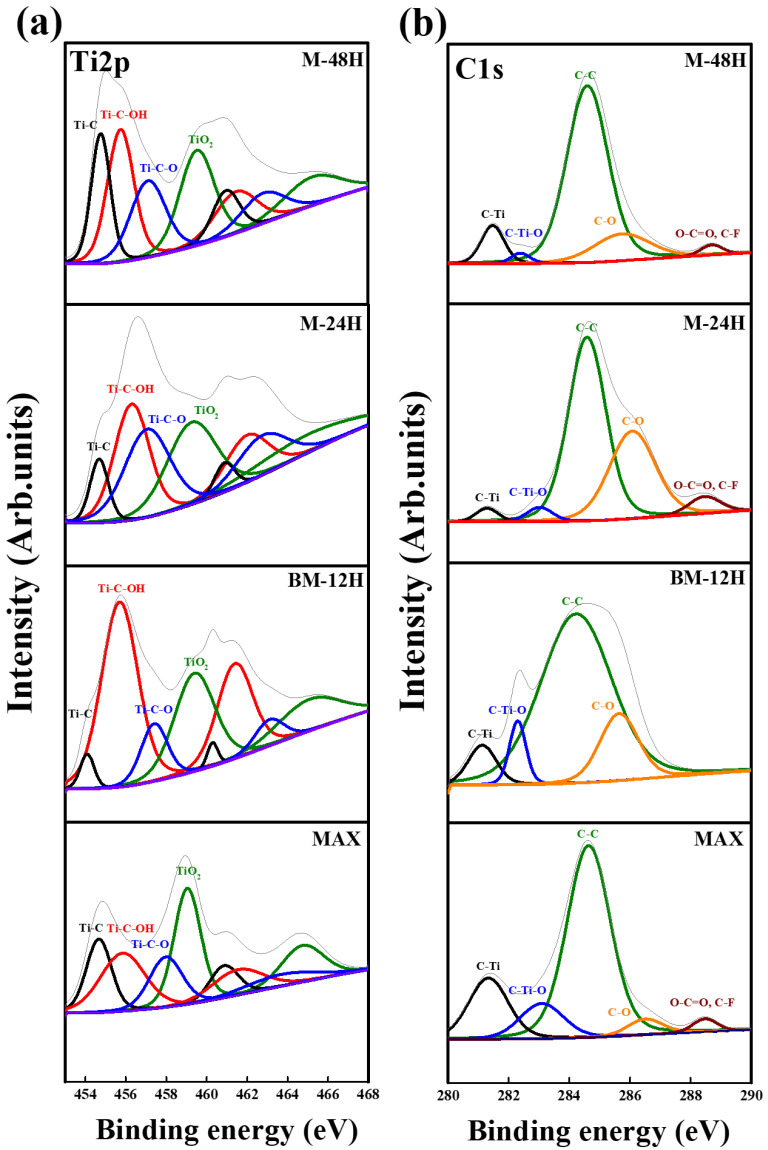
High-resolution XPS spectra of (**a**) Ti 2p spectrum and (**b**) C 1s spectrum of MXene sheets.

The surface oxidation state, fluorination and the surface chemical composition were investigated by high-resolution XPS spectra as shown in Figure 4. Figure 4a shows the deconvoluted Ti2p XPS spectra with a spin orbital splitting separation of 5.7 eV between Ti2p_3/2_: Ti2p _1/2_ and an area ratio of 1:2. Here, Ti2p has four bonds, Ti-C, Ti-C-OH, Ti-C-O and TiO_2_, located at the binding energies of 454.6, 455.9, 457.1 and 459.4 eV, respectively [65,66]. It was found that the HF solution-etched Ti_3_C_2_ MXene samples have -O, -OH and -F bond terminations at the surface [67]. Ti-C-OH and Ti-C-O bonds could have been formed due to the termination of functional groups. Hence, various binding energies related to Ti(I)-C-(O/OH/F), Ti(II)-C-(O/OH/F), Ti(III)-C-(O/OH/F) present at 455.0, 455.8, 457.2 eV, respectively [68]. It is very interesting to note that M-24H and BM-12H have higher Ti-C-OH content compared to Ti-C, which suggests high surface area due to the penetration of the -OH group in which the surface bond termination is easy. In particular, the BM-12H sample subjected to ball milling treatment has a higher -OH group content despite undergoing the shortest etching time. The XPS spectra of C1s are shown in Figure 4b and have four peaks corresponding to C-Ti, C-Ti-O, C-C, C-O and O-C=O, C-F bonds located at 281.3, 282.5, 284.4, 285.8 and 288.5 eV, respectively [32,69]. It is evident that sample BM-12H showed a high area ratio for the C-Ti-O bond compared to the C-C bond, suggesting a high surface area. In addition, peaks corresponding to the O-C=O and C-F bonds do not exist in sample BM-12H only, which means the work function of the sample can be lower due to the absence of the -F group termination [70]. This is also supported by the relatively low concentration of the F–C peak in sample BM-12H as shown Figure S3a [71,72,73,74]. Furthermore, in the BM-12H sample, the prominent Ti-C-OH bond (Figure 4a) and C-Ti-O bond (Figure 4b) have been formed due to the -OH group termination. It is also reported that the -OH group termination can lower the work function of the electrode material, thereby improving the electrochemical properties of the MXene sample [40,75,76,77,78]. This is supported by the highest concentration of the C-OH bond peaks in BM-12H as shown in Figure S3b [79,80].

### 3.4. Surface Area Analysis

**Figure 5:**
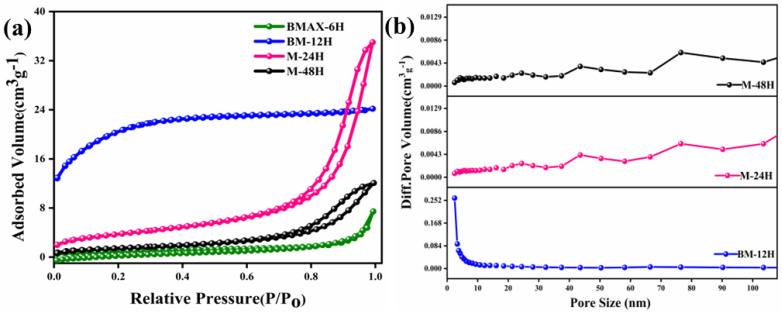
(**a**) N_2_ adsorption−desorption isotherms and (**b**) pore size distribution curves of the as-obtained MXenes.

The exfoliation of MAX phases and ball milling can enhance the specific surface area (SSA) and pore volume of MXenes. Hence, BET N_2_ adsorption−desorption was analyzed to study the surface area, pore volume, and pore size distribution (PSD) as shown in Figure 5a,b. All the non-ball-milled and etched MXene (M-24H, M-48H) samples (Figure 5a) exhibit practically vertical tails at relative pressures between 0.8 and 1.0, indicating the presence of macropores and negligible meso-porosity [37,60]. The N2 adsorption–desorption isotherms of sample BM-12H were found to be of Type I (Figure 5a), indicating the existence of microporosity. Interestingly, the ball-milled pretreated MAX-phase-derived MXenes (BM-12H) display a larger SSA (973.5 m^2^ g^−1^) and a pore volume of 0.2 cm^3^ g^−1^ (Table S3). Figure 5b shows the narrow and distinct PSD maximum centered at 8.7 nm which confirms the substantial increase in the micropore density in sample BM-12H. The samples M-24H and M-48H MXenes show the PSD maxima centered from around 60 to 80 nm, which clearly evidences the dominant macropore nature. This result reconfirms that the ball milling pretreatment processes produce porous MXenes with a much larger surface area than chemical etching processes [33,60].

### 3.5. Electrochemical Analysis

**Figure 6:**
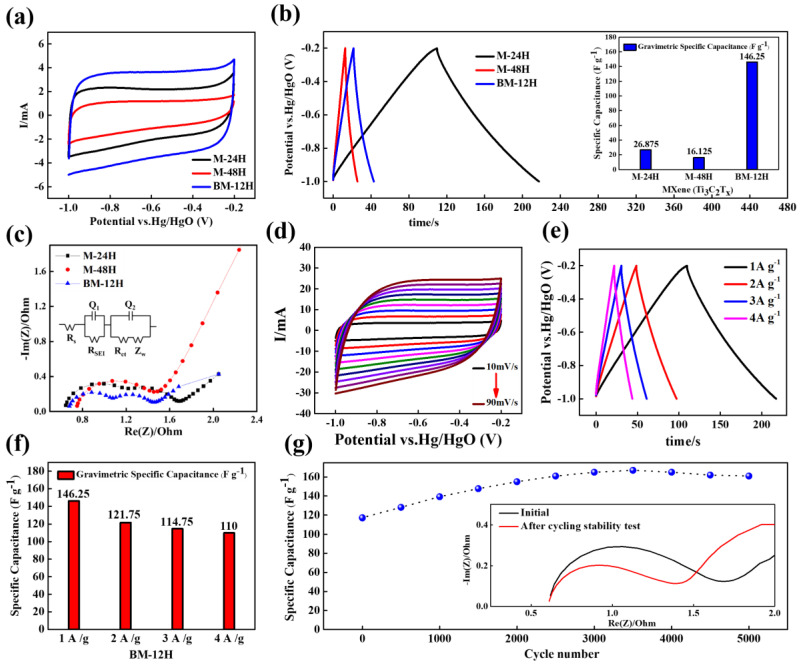
Comparison of (**a**) CV, (**b**) GCD curves (insert image: specific capacitance from GCDs), (**c**) Nyquist plots from the three-electrode system fitted by the equivalent circuit, (**d**) CV curves at various scan rates, (**e**) GCD curves at various current density, (**f**) specific capacitance at higher current density and (**g**) specific capacitance during the 5000-cycle charge-discharge stability test at 2 A g^−1^ (insert image: EIS of post-stability and stability-tested samples at high frequency) of BM-12H.

Figure 6 shows the CV, GCD, and Nyquist plots to investigate the electrochemical performance of the samples. The Figure 6a shows the CV curves of the samples performed at 10 mV s^−1^ in a potential window of −0.9 V to −0.2 V. All the samples show a nearly square shape, showing the characteristics of EDLC behavior, and among them, BM-12H shows the highest current value. This suggests the excellent electrochemical properties of sample BM-12H for supercapacitor electrode material [16,81]. Figure 6b shows the GCD conducted at a 1 A g^−1^ constant current which exhibits the linear and triangular characteristic of supercapacitor electrode materials [82,83,84]. The discharge time was longer for sample BM-12H, indicating the best specific capacitance nature. The performances are compared and shown in the inset figure by the bar graph. The specific capacitances of M-24H, M-48H and BM-12H were found to be 26.875, 16.125, and 146.25 F g^−1^, respectively, from the GCD curve. A comparison of specific capacitance with other publications can be found in Table S2. In addition, the M-12H sample without ball milling pretreatment showed poor CV performance at a current density of 1 A g^−1^. This suggests that without ball milling pretreatment, a 12 h HF treatment may not be sufficient. The CV and GCD results of samples M-12H and M-24H are shown in Figure S4.

Figure 6c shows the equivalent circuit model used for EIS fitting. The fitted EIS parameters are shown at Table S4. The BM-12H sample exhibits significantly lower RSEI (0.44 Ω) and Rct (0.33 Ω) than other samples, indicating much better charge transport [85]. The equivalent series resistance (Rs) could be obtained from the Nyquist plot *x*-axis intercept at high frequency. The value of Rs is very low; it is 0.64, 0.74, 0.67 Ω for BM-12H, M-48H and M-24H, respectively; however, M-48H has a slightly higher value. Moreover, the low frequency slope is significantly higher for M-48H than M-24H and BM-12H, indicating a relatively higher ion diffusion resistance than the other two samples. This supports the low electrochemical performance of M-48H [86]. In addition, BM-12H has the lowest Zw value of 1.31 Ωs^−1/2^ which suggests that the sample may have better electrolyte permeability than M-48H (9.12 Ωs^−1/2^) and M-24H (1.63 Ωs^−1/2^) during electrochemical analysis [87].

Figure 6d shows the CV curve at various scan rates ranging from 10 to 90 mVs^−1^ for sample BM-12H, which again reaffirms the EDLC behavior even at the 90 mV scan rate and indicates the material’s stability. The GCD curves at various current densities (1–4 A g^−1^) are shown in Figure 6e for sample BM-12H. The charge–discharge graph is consistently linear at all current densities and the IR drop is negligibly small, indicating an almost perfect symmetrical triangular graph.

Figure 6f shows the change in the specific capacitance at different current density for sample BM-12H and exhibits a good capacitance of 110 F g^−1^ with a retention rate of 75% at very high current density. Figure 6g shows the change in specific capacitance during the 5000-cycle charge–discharge test conducted at 2 A g^−1^ for sample BM-12H. The charge–discharge cycle graph shows an increase in specific capacitance due to the termination of the -OH group, intercalation of the K^+^ ion and transformation to the TiO_2_/Ti_3_C_2_ hybrid structure as shown at the XPS spectra of stability tested samples in Figure S5, which agrees with our previous study [52]. Figure 6g inset shows the Nyquist plots for the pristine electrode that underwent the charge–discharge cycling test. The Rs values were found to be almost unchanged, whereas Rct decreased from 1.0 Ω and 0.7 Ω after the cycling test. It suggests an efficient electrolyte penetration and enhanced interaction of the electrolyte/electrode surface which caused increased specific capacitance after the cycling test.

### 3.6. Fabrication of Symmetrical Coin Cell

**Figure 7:**
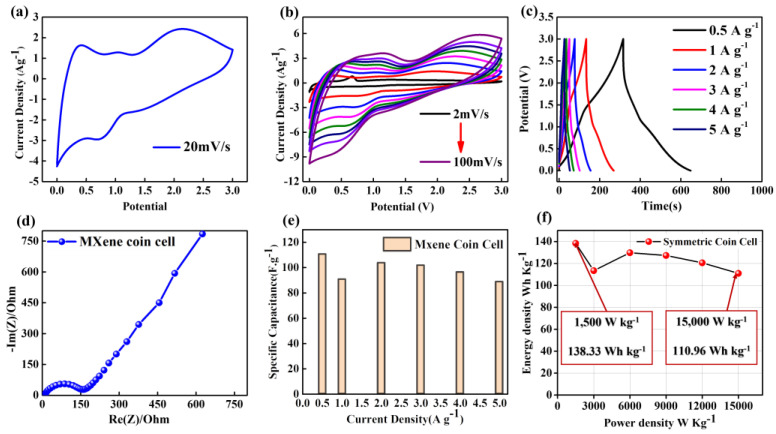
Electrochemical behavior of BM−12H symmetric coin cell using 1 M LiPF_6_ organic electrolyte. CV at (**a**) 20 mVs^−1^ and (**b**) various scan rates, (**c**) GCD at different current densities (inset at a current density of 0.5 A g^−1^), (**d**) Nyquist plots (inset: high-frequency region), (**e**) specific capacitances at different current densities and (**f**) Ragone plot of symmetric coin cell SC device.

Since the aqueous electrolyte is limited to 1 V, the electrochemical characterizations of MXene electrodes were performed in an organic electrolyte in order to extend the potential window up to 3 V to realize its real practical applications [88]. The supercapacitor coin cell was fabricated using BM-12H electrodes with a 1 M LiPF_6_ organic electrolyte [89]. The excellent EDLC properties of MXene materials were confirmed in the KOH electrolyte, whereas pseudocapacitance properties were also realized in the LiPF_6_ electrolyte. Figure 7a shows the CV plot at a scan rate of 20 mVs^−1^ in the potential window range of 0 to 3 V. A set of redox peaks around 0.4 V to 0.7 V is clearly seen due to the existence of the intercalation pseudocapacitance charge storage caused by the intercalation/deintercalation of Li^+^ between the sheets of 2D MXenes [90,91]. Figure 7b shows the CV profiles at different scan rates ranging from 2 to 100 mVs^−1^, which still demonstrates the same redox behavior originating from the intercalation pseudocapacitive properties of MXenes [90,92]. Figure 7c presents the GCD curves of coin cell SC at different current densities; it exhibits a triangular shape with a slight distortion due to the high reversibility of the redox reactions caused by the intercalation pseudocapacitance [91,92], which agrees with the results of the CV curves. Figure 7d shows the EIS of the symmetrical MXene SC coin cell with an Rs equivalent to 3.8 Ω, and a vertical line (Warburg Tail) between 60° and 45° to the real axis indicates a pseudocapacitive behavior of MXene sheets [91,93]. The gravimetric capacitances calculated by GCD as a function of the current densities are plotted in Figure 7e. Obviously, the MXene coin cell has the highest areal capacitance of 110.6 F g^−1^ at 0.5 A g^−1^ and could still achieve a high capacitance value of 88.7 F g^−1^ even at a higher current density of 5 A g^−1^ with a retention ratio of 80%, showing good capacitive behavior and rate capability of multilayered MXene sheets. The Ragone plot (Figure 7f) shows the highest energy densities of 138.3 and 111 Wh kg^−1^ at power densities of 1500 and 15,000 W kg^−1^, respectively, compared to symmetrical supercapacitors due to the effect of both the EDLC and pseudocapacitance characteristics [88,94,95,96]. This result shows the superiority of Ti_3_C_2_ as a symmetric supercapacitor electrode material [97]. The MXene coin cell SC electrode’s cyclic stability (Figure S2) was examined for 6000 charge–discharge cycles at a constant current density of 5 A g^−1^, and very impressive capacity retention of 90% was recorded, which is extremely encouraging for their long-term durable use.

## 4. Conclusions

In summary, chemical etching combined with ball milling is a simple and rapid technique for a large-scale production of 2D layered MXenes. Herein, we envisioned the mechanical milling method that stimulated the Al exfoliation and the structural change in the MAX phase bulk materials. Benefitting from the wider interlayer distance as well as the 2D sheet-like morphology, BM-12H exhibits high specific capacitance of 146.2 F g^−1^ at 1 A g^−1^ and excellent cyclability, far ahead of non-ball milled MXene sheets. In addition, the symmetric coin cell electrode with a 1 M LiPF_6_ organic electrolyte exhibited an energy density of 138.3 W h kg^−1^ at a power density of 1500 W kg^−1^. This work highlights the significance of the chemical etching combined mechanical milling technique and discusses the corresponding microstructure and physical properties to aid in the creation of high-performance Mxenes.

## Figures and Tables

**Table 1:** Comparison of D-spacing [Å] and width of lattice pattern.

	(103)		(105)		(110)	
	D-Spacing (Å)	Width of Lattice Pattern (nm)	D-Spacing (Å)	Width of Lattice Pattern (nm)	D-Spacing (Å)	Width of Lattice Pattern (nm)
M-48H	2.42	0.24	2.100	0.211	1.489	-
M-24H	2.46	0.24	2.175	0.213	1.493	-
BM-12H	2.5	0.25	2.224	0.219	1.512	-

## Data Availability

The data presented in this study are available within the article.

## Supplementary Materials

The following supporting information can be downloaded at: https://www.mdpi.com/article/10.3390/nano13111741/s1. References [98,99,100,101] are cited in the Supplementary Materials.
