# Supplementary material for: Mechanochemical Pretreated Mn+1AXn (MAX) Phase to Synthesize 2D-Ti3C2Tx MXene Sheets for High-Performance Supercapacitors
Source: Nanomaterials (Basel). 2023 May 26;13(11):1741. doi: 10.3390/nano13111741 (PMC10254144; doi:10.3390/nano13111741)
Supplement: Supplementary file 1 [file nanomaterials-13-01741-s001.zip › nanomaterials-2398025-supplementary.pdf]

Supplementary Materials

Table S1. Synthesis details of 2D layered MXenes from MAX phases.

**S. No**	**Synthesis Process**<br>**(Ball Milling+ Chemical Etching)**	**Abbreviation**
**1**	6 h Ball Milled MAX phase	**BMAX-6H**
**2**	6 h Ball milled and 12 h etched and dried at 60°C	**BM-12H**
**3**	12h etched and dried at 60°C	**M-12H**
**4**	24h etched and dried at 60°C	**M-24H**
**5**	48h etched and dried at 60°C	**M-48H**
Etching By HF.		

Calculation of electrochemical parameters:

The electrochemical parameters, such as the specific capacitance (Cs, Fg−1), Coulombic efficiency (η), energy (E), and power density (P) are important parameters for the investigation of the capacitive behavior of electrochemical cells.

Calculation of specific capacitance for three electrode system:

In order to evaluate the performances of the cells, the specific capacitances for the electrodes were tested in three-electrode system. The specific gravimetric capacitance (Fg−1) for the three-electrode setup was calculated from galvanostatic charging and discharging (GCD) curves using the equation 1:

$$C_{sc}(F\,g^{-1}) = \frac{I\Delta t}{\Delta V m} \qquad (1)$$

where, I is the discharge (or charge) current, Δt is the discharge (or charge) time, ΔV is the potential window of cycling, and m is the mass loaded on carbon cloth substrate.

Specific capacitance for two electrode cell:

The gravimetric capacitance for symmetric supercapacitor was calculated from galvanostatic charge/discharge curves using the equation 2:

$$CSC(F\,g^{-1}) = \frac{2I\Delta t}{\Delta V m} \qquad (2)$$

where Csc (F g-1) is the gravimetric specific capacitance, i (A) is the discharge current, Δt (s) is the discharge time, ΔV is the potential window (excluding the IR drop), and m in gram is the mass of the active material.

Power and energy density calculations:

The power density (P) of the symmetric supercapacitor was calculated from the energy density (E) and the discharge time (t) according to the Equations (3) and (4)

$$E_{cell} = \frac{1}{2} C_{sc} \Delta V^2 . \frac{1}{4 * 3.6} \qquad (3)$$

$$P_{cell} = \frac{E_{cell}}{\Delta t} \qquad (4)$$

where E is the energy density (Wh kg−1), C is the measured device capacitance (Fg−1) calculated using eq (2), V is the potential window (V), P is the power density (W kg−1), and Δt is the discharge (or charge) time (s) from the CD curves.

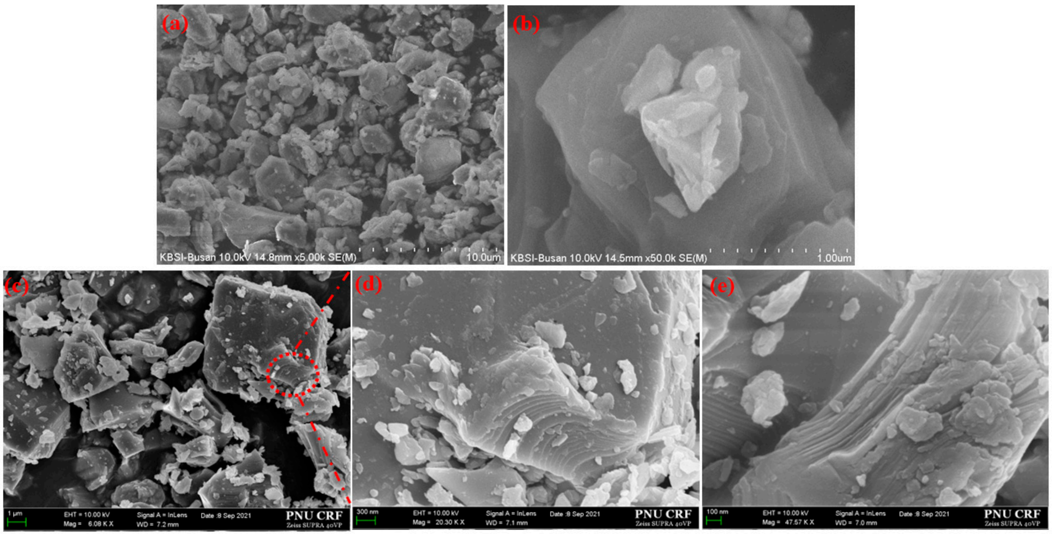

Figure S1. FESEM images of (a and b) pristine Ti_3AlC_2, (c, d, e) Ball milled MAX phase at low and high magnification.

Table S2. The comparison of MXene electrode supercapacitor's various parameters in 3 electrode system with aqueous electrolyte.

No.	Material	Electrolyte	Potential Window(V)	Specific Capacitance (F g^{-1})	Ref
1	Ti_3C_2	1M NaCl	1.2	127.6 at 0.1A g^{-1}	[1]
2	Ti_3C_2/TiO_2-nanowire	6M KOH	0.6	135 at 5mV/s	[2]
3	Ti_3C_2	0.5M NaCl	1.2	132 at 0.1 A g^{-1}	[3]
4	Ti_3C_2	1M NaCl	0.9	75.76 at 0.1A g^{-1}	[4]
5	Ti_3C_2	1M H_2SO_4	0.5	105.8 at 1A g^{-1}	[5]
6	Ti_3C_2	3M KOH	0.8	141.45 at 0.4A g^{-1}	[6]
7	Ti_3C_2	3M KOH	0.8	146.25 at 0.5Ag-1	This work

Table S3. Surface Area, Pore Size, and Pore Volume Data.

MXene Samples	SSA (m^2 g^{-1}) [a]	Pore Volume (cm^3g^{-1}) [a]	Pore diameter (nm)[a]
BMAX-6H	8.57	0.02	-
BM-12H	32.8	0.24	7.6
M-24H	23.7	0.13	22.1
M-48H	17.28	0.08	24.8

Table S4. The impedance parameters derived by equivalent circuit model.

**Electrode**	Rs(Ω)	R_{SEI}(Ω)	Q_1(μF)	R_{ct}(Ω)	Q_2(μF)	Z_w(Ω $s^{-1/2}$)
M-24H	0.64	0.56	23.23	0.43	335	1.63
M-48H	0.74	8.68	5670	0.65	24.23	9.12
BM-12H	0.7	0.40	27.01	0.33	668	1.31

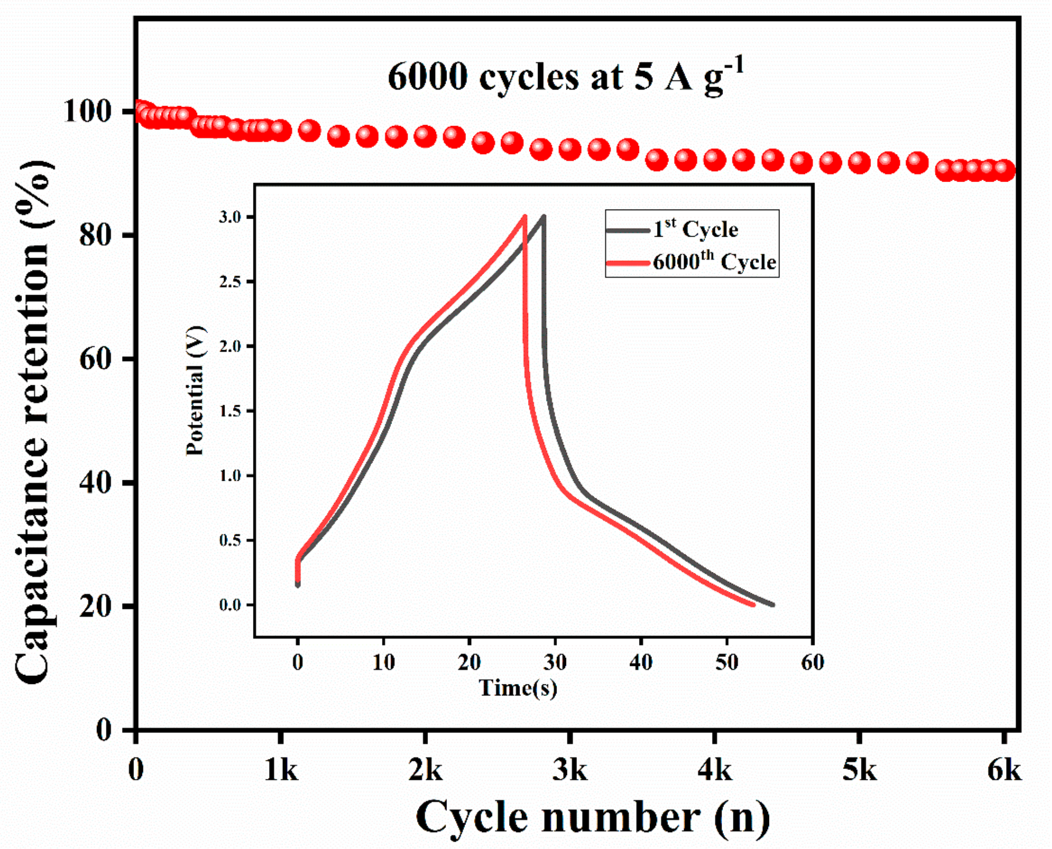

Figure S2. Cyclic stability of MXene coin cell at current density of 5 Ag^{-1}.

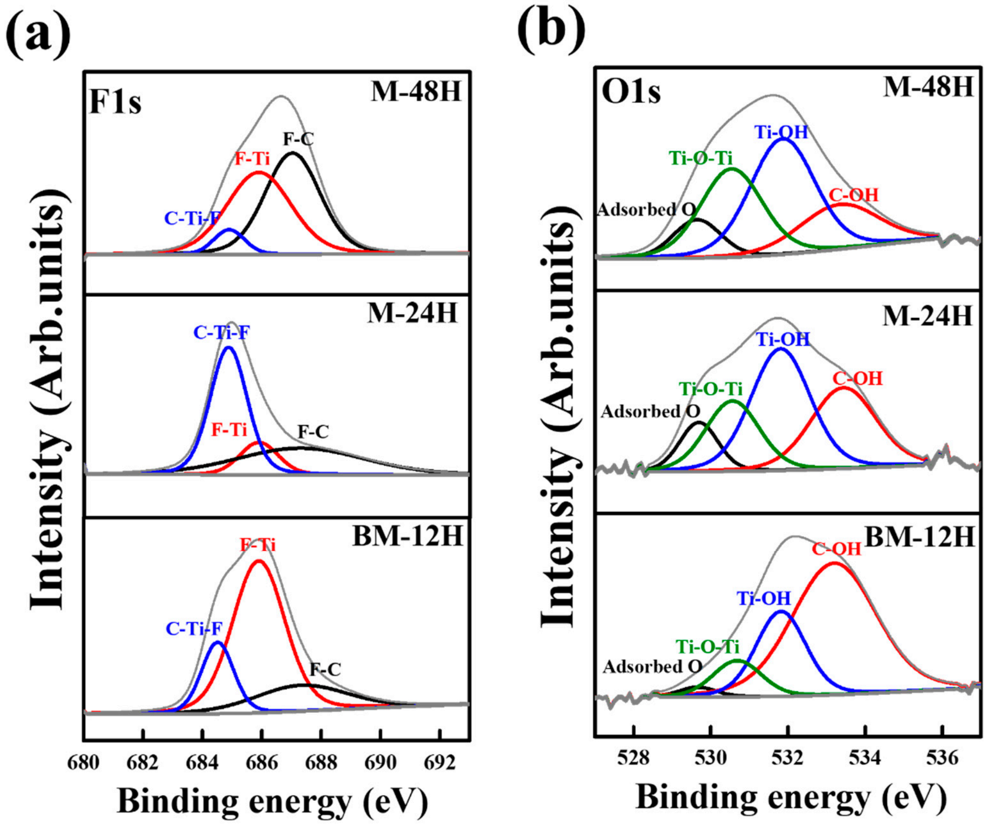

Figure S3. XPS fitting of (a)F1s and (b)O1s for M-48H, M-24, BM-12H.

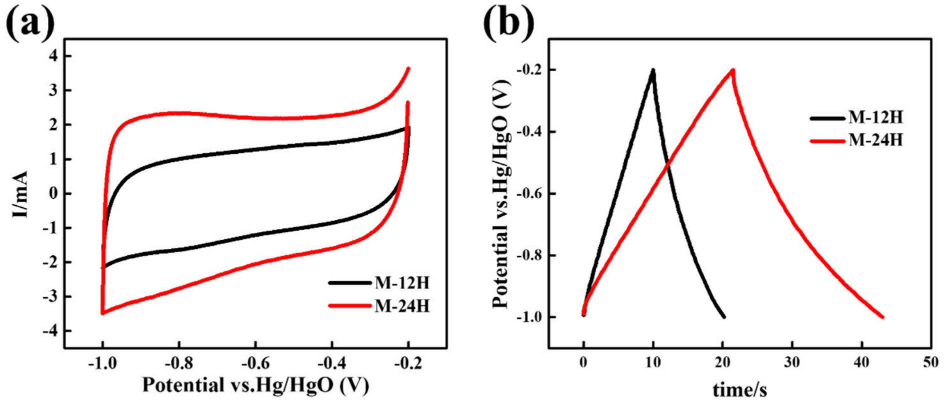

Figure S4. Comparison in (a) CV and (b) GCD for M-12H and M-24H.

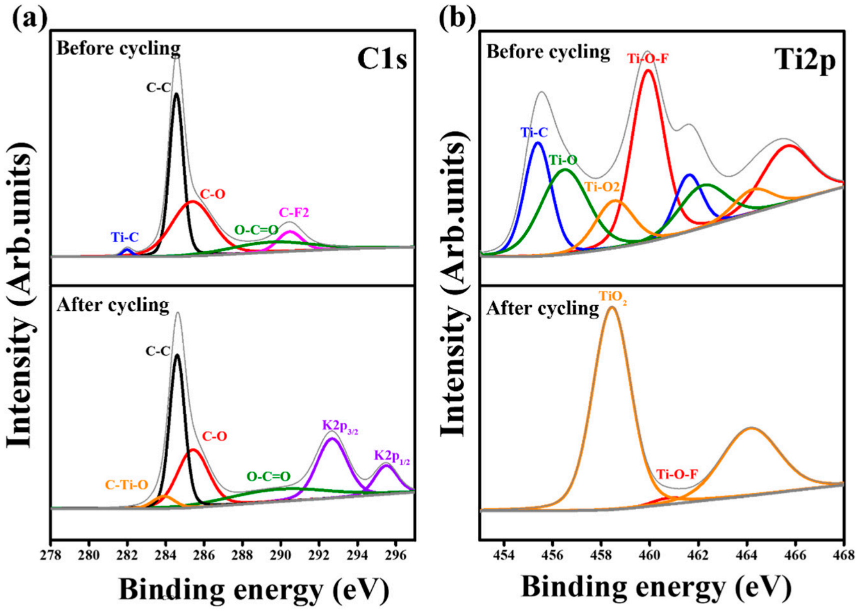

Figure S5. Comparison of XPS spectra in (a)C1s and (b)Ti2p before and after cycling tested BM-12H electrode.

References

1. Ma, J.; Cheng, Y.; Wang, L.; Dai, X.; Yu, F. Free-Standing Ti3C2Tx MXene Film as Binder-Free Electrode in Capacitive Deionization with an Ultrahigh Desalination Capacity. *Chemical Engineering Journal* **2020**, *384*, doi:10.1016/j.cej.2019.123329.
2. Cao, M.; Wang, F.; Wang, L.; Wu, W.; Lv, W.; Zhu, J. Room Temperature Oxidation of Ti_3C_2 MXene for Supercapacitor Electrodes. *J Electrochem Soc* **2017**, *164*, A3933–A3942, doi:10.1149/2.1541714jes.
3. Srimuk, P.; Kaasik, F.; Krüner, B.; Tolosa, A.; Fleischmann, S.; Jäckel, N.; Tekeli, M.C.; Aslan, M.; Suss, M.E.; Presser, V. MXene as a Novel Intercalation-Type Pseudocapacitive Cathode and Anode for Capacitive Deionization. *J Mater Chem A Mater* **2016**, *4*, 18265–18271, doi:10.1039/c6ta07833h.
4. Buczek, S.; Barsoum, M.L.; Uzun, S.; Kurra, N.; Andris, R.; Pomerantseva, E.; Mahmoud, K.A.; Gogotsi, Y. Rational Design of Titanium Carbide MXene Electrode Architectures for Hybrid Capacitive Deionization. *Energy and Environmental Materials* **2020**, *3*, 398–404, doi:10.1002/eem2.12110.
5. Hou, W.; Sun, Y.; Zhang, Y.; Wang, T.; Wu, L.; Du, Y.; Zhong, W. Mixed-Dimensional Heterostructure of Few-Layer MXene Based Vertical Aligned MoS2 Nanosheets for Enhanced Supercapacitor Performance. *J Alloys Compd* **2021**, *859*, doi:10.1016/j.jallcom.2020.157797.
6. Cho, I.; Selvaraj, A.R.; Bak, J.; Kim, H.; Prabakar, K. Anomalous Increase in Specific Capacitance in MXene during Galvanostatic Cycling Studies. *J Energy Storage* **2022**, *53*, 105207, doi:10.1016/j.est.2022.105207.
